# Virtual Reality in Palliative Care: A Systematic Review

**DOI:** 10.3390/healthcare10071222

**Published:** 2022-06-29

**Authors:** Jessica L. Martin, Dimitrios Saredakis, Amanda D. Hutchinson, Gregory B. Crawford, Tobias Loetscher

**Affiliations:** 1 UniSA Justice & Society, University of South Australia, Adelaide 5001, Australia; marjl019@mymail.unisa.edu.au (J.L.M.); amanda.hutchinson@unisa.edu.au (A.D.H.); tobias.loetscher@unisa.edu.au (T.L.); 2 Northern Adelaide Local Health Network, Adelaide 5092, Australia; gregory.crawford@adelaide.edu.au; 3 Discipline of Medicine, University of Adelaide, Adelaide 5005, Australia

**Keywords:** head-mounted display, hospice, palliative care, virtual reality

## Abstract

Background: Virtual reality (VR) using head-mounted displays (HMDs) has demonstrated to be an effective tool for treating various somatic and psychological symptoms. Technological advances and increased affordability of VR technology provide an interesting option for delivering psychological interventions to patients in palliative care. The primary aim of this systematic review was to synthesise the available research on the use of VR for enhancing psychological and somatic outcomes for palliative care patients. Secondary aims included assessing general satisfaction and overall usability. Method: A pre-registered systematic literature search was conducted according to PRISMA guidelines using OVID Emcare, Cochrane Library, Embase, Medline, PsycINFO, and PubMed Care Search: Palliative Care Knowledge Network. Peer-reviewed experimental, quasi-experimental, observational, case, and feasibility studies consisting of single or multiple VR sessions using HMDs that reported psychological and/or somatic outcomes were included. Results: Eight studies published between 2019 and 2021 were included, representing 138 patients. While the reported quantitative psychological and somatic outcomes were ambiguous, the qualitative outcomes were largely positive. Participants were generally satisfied with VR, and most studies reported the VR interventions as usable, feasible, and acceptable. Conclusions: VR shows promise in palliative care and generally addresses a range of symptoms with few adverse effects. Future research should consist of adequately powered RCTs evaluating dosage and focusing on providing meaningful activities to enhance outcomes further.

## 1. Introduction

Fully immersive Virtual Reality (VR) using head-mounted displays (HMDs) is a powerful technology increasingly used in a wide range of health care settings [1,2,3,4]. Evidence shows that VR interventions can alleviate pain symptoms and distract patients during medical procedures [5,6,7]. This evidence makes VR technology an interesting option for delivering psychological interventions in a palliative care setting.

Psychological interventions are an important aspect of providing a holistic approach to palliative care [8]. One approach to improving palliative care patients’ psychological well-being is to focus on delivering meaningful and tailored activities. It has been shown that palliative care patients want to engage in meaningful activities related to something significant throughout their lives [9]. Participation in meaningful activities may help palliative care patients maintain dignity and quality of life [10]. However, barriers to providing meaningful activities include time, financial resources, health status, and fatigue [10,11]. VR may assist with overcoming barriers to providing meaningful psychological interventions.

The use of VR as a tool for therapy has been shown to help facilitate meaningful activities in older adults [12]. Furthermore, VR has also been efficacious in enhancing psychological outcomes and treating psychological disorders, including anxiety, depression, and psychosis [13]. VR in the form of HMDs provides an immersive experience where the user views a stereoscopic image that can be updated in real-time with movement and can provide a feeling of “being there” in the virtual environment [14]. This realistic experience that VR provides can be used bedside [15], tailored to an individual using readily available applications [16,17], and may assist with increasing engagement in therapy [18]. Using VR for meaningful activities may help to encourage engagement further and overcome barriers associated with psychological interventions for palliative care patients. Although VR is a promising intervention across a range of therapeutic contexts, its use has also been associated with negative side effects.

The collection of side effects commonly observed while using VR has been labelled “cybersickness” and is generally reported to consist of symptoms including nausea, dizziness, disorientation, light-headedness, and headaches [19,20]. The prevalence of nausea in palliative care cancer patients has been reported as high as 68% [21]. Therefore, greater care must be taken when using VR with palliative care patients so as not to exacerbate any existing symptoms. Despite the risk of side effects, the role that VR technology can play in facilitating palliative care may outweigh the negative effects.

While the use of VR specifically in palliative care is an emerging area, recent research investigating how VR can be used to improve physical and psychological well-being has demonstrated promising results [22,23]. To increase our understanding of VR use in palliative care patients, a comprehensive review and analysis of the available data are required. There is also a need to examine the acceptance and usability of VR in palliative care patients.

The primary aim of this systematic review is to synthesise the available research on the use of VR for enhancing psychological and somatic outcomes for palliative care patients. Secondary aims include assessing general satisfaction and overall usability of VR for palliative care patients. 

## 2. Materials and Methods

The Preferred Reporting Items for Systematic Review and Meta-Analysis (PRISMA) guidelines were followed when conducting this systematic review [24].

### 2.1. Protocol and Registration

Following the Preferred Reporting Items for Systematic Review and Meta-Analysis Protocols [25], a study protocol was pre-registered via Open Science Framework (doi:10.17605/OSF.IO/5JQUX). Some deviations from the protocol were necessary. Considerations around content, duration/frequency, and technology were added to this review, and a GRADE rating was not included. A meta-analysis was not possible due to the small number of studies and the heterogeneity of the measures. 

### 2.2. Eligibility Criteria

This review included palliative care patients engaged in VR interventions using HMDs. The WHO definition of palliative care was used [26]. This definition describes palliative care as an approach that “improves the quality of life of patients and that of their families who are facing challenges associated with life-threatening illness, whether physical, psychological, social or spiritual” [26]. Participants needed to be defined as “in palliative care” or as “living with a life-limiting illness”. Peer-reviewed experimental, quasi-experimental, observational, case, and feasibility studies consisting of single or multiple VR sessions were included. Studies also needed to report psychological and/or somatic outcomes. No restrictions on language or year of publication were imposed.

### 2.3. Information Sources/Search Strategy

A broad, systematic literature search was carried out in August 2021 in the following electronic databases: OVID Emcare, Cochrane Library, Embase, Medline, PsycINFO, and PubMed Care Search: Palliative Care Knowledge Network. Results from the systematic search were forward and backward snowballed via Google Scholar using the reference list and citations of the articles to identify additional articles. Search terms were adjusted when necessary depending on the database. The search terms that were used were:

1. Virtual reality OR virtual environment* OR VR OR VR headset OR virtual reality headset OR head-mounted display OR HMD OR helmet-mounted display
2. Palliat* OR hospice OR end of life OR terminal care OR life support care OR terminally ill OR terminal-stage* OR advanced disease OR (“end-stage disease*” or “end-stage disease* or end-stage illness” or “end-stage”) OR last year of life OR life’s end
3. 1 AND 2

### 2.4. Data Management

Literature search results were uploaded to Covidence, an internet-based program for systematic review management [27]. Full texts of studies that were accepted after the title and abstract screening phase had the citation, abstract and full text uploaded to Covidence. 

### 2.5. Selection Process

Two authors (JLM and either DS or TL) independently screened titles and abstracts of records found. Studies that meet the inclusion criteria had the full text screened, and a third author was consulted for cases that resulted in disagreement between the first two reviewers. For full texts, the reason for excluding the trial was recorded. 

### 2.6. Data Collection Process and Data Items

Data extraction templates were created using the headings Author, Year of Publication, Quality Score, Aims/Objectives, Setting, Patients, VR Technology, Intervention, Outcome Measures, Findings, Negative Effects, Conclusion, and Miscellaneous Notes. Data extraction from articles was conducted by two authors (JLM and DS). 

### 2.7. Study Risk of Bias Assessment

We assessed and reported the methodological risk of bias in the included studies in accordance with the Joanna Briggs Critical Appraisal Tools for Quasi-Experimental Studies [28]. We judged each item as high, low, or with some concerns regarding the risk of bias as set out by criteria used previously by Macnamara et al. [29]. Using this approach, studies were classed on their risk of bias by the percentage of ‘yes’ criteria met, where low risk indicated >70%, 50–69% indicated moderate risk, and <49% indicated high risk. Two authors (JLM and DS) independently assessed the risk of bias in included studies, and any disagreements were resolved through discussion with a third author (TL).

### 2.8. Outcomes 

The primary measures of interest were psychological (including anxiety, depression, quality of life, and overall psychological well-being) and somatic (including pain, fatigue, and analgesic use) outcomes. Secondary outcomes included overall patient satisfaction with VR, barriers to implementation, and overall usability.

### 2.9. Synthesis Methods

A meta-analysis was not possible due to the heterogeneity of the data.

#### Narrative Synthesis

A systematic synthesis is provided with information to summarise and explain the findings of the included studies. This synthesis explores the findings, along with the relationship both within and between the included studies.

## 3. Results

**Figure 1:**
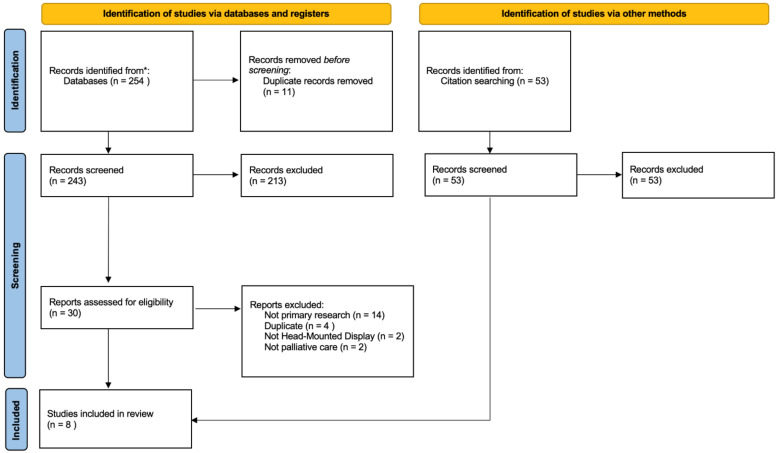
PRISMA Search Flow Diagram.

In total, 243 studies were identified following the database search, and 53 were identified through citation searching. After removing duplicates and following the title and abstract screening, 30 studies progressed to the full-text review. Following this, eight studies were included. No additional records were found through citation searching. See Figure 1 for the 2020 PRISMA flow diagram of the article screening process.

The demographic and setting information, study aims, details regarding the VR technology, and intervention of all studies are summarised in Table 1. Key findings are presented in Table 2.

### 3.1. Risk of Bias Assessment

The risk of bias assessment revealed several threats to internal validity. Of the eight included studies, four were assessed as having a high risk of bias, three were judged as having a moderate risk of bias, and only one had a low risk of bias. Table 3 contains information regarding the risk of bias for each study. Furthermore, approximately 14% of responses were unclear as insufficient methodological information was provided to make an informed judgment about the risk of bias. 

### 3.2. The Efficacy of VR Interventions in Palliative Care Patients Regarding Psychological Outcomes and Somatic Outcomes

Three studies [23,30,31] utilised versions of the Edmonton Symptom Assessment Scale (ESAS) to measure the change in symptom burden in participants before and after VR intervention. Johnson et al. [30] found an improvement in appetite based on a 95% confidence interval (*p =* 0.279). The authors also noted that there appeared to be an overall trend of symptom improvement after the VR intervention in terms of pain, tiredness, drowsiness, depression, and anxiety. Niki et al. [23] found overall statistically significant improvements (*p =* <0.05) in pain, tiredness, drowsiness, shortness of breath, depression, anxiety, well-being, fun, and happiness. Further statistical analysis separated patients into two groups: those who had visited a memorable place and those who had visited a place they had always desired to go to but never visited. The significant improvements in the aforementioned symptoms, plus a statistically significant improvement in lack of appetite, were reported in patients who had visited a memorable place, with the largest effect size observed for depression (*d* = 1.237). There were no significant improvements in any symptoms in the group that visited new or desired places. Perna et al. [31] compared pre and post-ESAS scores in those who received a personalised VR intervention with those who received a non-personalised intervention. No overall statistical differences were found; however, it was reported that those in the personalised group appeared to experience the greatest improvement in tiredness, anxiety, and psychological well-being, while those in the non-personalised group appeared to experience the greatest improvements in tiredness and drowsiness using mean difference scores. Other studies qualitatively reported an improvement in psychological [22,32,33,34] and somatic outcomes [32,33,34]. 

### 3.3. General Satisfaction with VR

Generally, most participants were satisfied with the VR interventions they were offered. Three themes were identified relating to palliative care patients’ satisfaction with VR: the experience [22,23,32,33,34,35], overall likeability of the intervention [22,30,32,34,35] and perceived benefit and helpfulness [22,30,32,33].

### 3.4. Experience 

Qualitative data from 14 participants in Brungardt et al. [34] reported the VR intervention as comfortable and easy to engage with; however, two participants found the experience acceptable but did not enjoy it. Similarly, Lloyd and Haraldsdottir [32] found that most participants described their experience with the VR intervention positively, including reports of joy and happiness and “being lifted out of their current situation” (p348). In addition, some neutral responses and very little negative feedback were provided. Ferguson et al. [35] identified four themes relating to the perceived experience of VR use. First, *narration* when a participant retold what they experienced (44% of participants narrated their experiences by saying things such as “I see blue and the sun”, and “The sounds of the ocean, this is really wonderful, I love the ocean”). Second, *affirmation* (44% of participants reported they either enjoyed or appreciated the VR experience). Third, *comfort level* (50% of participants reported on comfort level and included both positive and negative comments about wearing the headset, for example, “head was too small for the strap”, wanting headset removed, and “so comfortable…so soothing”). Finally, *unfulfilled* (statements like “is this all I’m going to see?”). The study by Niki et al. [23] found a better experience in those who visited a memorable place than those who visited desired or new places indicated by expectations measured post-VR. A majority of participants, 93% (14/15) in Nwosu et al. [22], reported the VR experience as positive and would all like to use VR again. Finally, a case study by Weingarten et al. [33] found the experience new and exciting. 

**Table 1:** Virtual reality in palliative care studies: demographics, setting, VR technology, aims, intervention, and number of sessions/duration/frequency.

Studies and Year of Publication	Demographics		Setting	Population	VR Technology	Aims	Intervention	Number of Sessions (Duration/Frequency)
	*N* (M/F)	Mean Age (SD)						
Brungardt et al., 2020 [34]	23 (11/12)	47.4 (17.1)	Hospital, U.S.A.	Hospitalised adults (18+ years) with a palliative care consult.	Oculus Go	To evaluate implementation measures of feasibility, usability, and acceptability of a VR-based music therapy intervention.	Patient created customised soundtrack to listen to during one of four nature-based 360° VR environments	1 (<30 min)
Ferguson et al., 2020 [35]	25 (3/22)	85 (8.9)	Hospice, U.S.A.	Convenience sample with diagnosis of dementia.	Mirage Solo	To explore acceptability, tolerability, and subjective experience of VR as therapeutic recreation for hospice patients living with dementia.	Pre-selected VR experience. YouTube VR 360 beach scene video	1 (~35 min)–3.5 min video looped for up to 12 times, 12.4 min average.
Johnson et al., 2020 [30]	12 (4/8)	72 (16)	Hospice, U.S.A.	Patients with life-limiting illness.	Samsung Gear	To examine the utility of VR for palliative care patients	Pre-selected VR experience using one of nine low-cost, easy-to-use applications (e.g., “360 Photos”, “Meditation,” “Hello Mars.”)	1 (30 min, 11/12 participants at least 20 min)
Lloyd & Haraldsdottir, 2021 [32]	19 (10/9)	69.6 (15.4)	Hospice, U.K.	Adult inpatients and outpatients diagnosed with a life-limiting condition.	Not reported	To explore the acceptability and potential benefits of using immersive VR for people with life-limiting conditions in a hospice setting.	Personalised VR experience. Participants asked to decide on a destination of choice.	1 (30 min)
Niki et al., 2019 [23]	20 (14/6)	72.3 (11.9)	Palliative care wards, Japan.	Patients (20+ years) with a terminal cancer diagnosis.	HTC VIVE	To verify whether simulated travel using VR is efficacious in improving symptoms in terminal cancer patients.	Personalised VR experience. Participants asked where they wanted to go using Google Earth VR^®^.	1 (~30 min)
Nwosu et al., 2021 [22]	15 [12 patients, 3 caregivers] (9/6) 7 staff members 6 representatives	63 [median] (16.5)	Hospital and hospice, U.K.	Inpatients and outpatients from both units Staff from both units. Members of the general public for evaluation	Samsung Gear	To explore the feasibility of implementing VR therapy for patients and caregivers in a hospital specialised inpatient palliative care unit and hospice and to identify questions for organisations to support VR adoption in palliative care.	Pre-selected VR experience from one of three applications: guided relaxation video of a beach, guided meditation through forest, or rollercoaster ride.	1 (5–10 min)
Perna et al., 2021 [31]	26 (12/14)	Range 27–85	Hospice, U.K.	Patients under hospice care (18+ years) with progressive life-limiting illness.	Google Daydream	To test the feasibility and acceptability of recruiting people with advanced illness into a trial with multiple VR sessions and to determine whether outcomes on the ESAS show any effect of personalised VR.	Participants randomised into 2 groups: personalised and pre-selected VR experience. Personalised group participated in an interview to obtain preferences for VR sessions, and non-personalised group offered a randomly selected VR session from a set of 6 pre-selected experiences.	4 (4 min, once weekly)
Weingarten et al., 2019 [33]	1 (0/1)	12	Hospital, Canada.	One patient with myelocytic leukaemia.	Not reported	To trial VR program as a part of therapeutic supports to inform a future pilot project.	Personalised VR experience tailored to the patients’ specific wants and needs.	1 (5–10 min)

**Table 2:** Virtual Reality in Palliative Care: Primary and Secondary Outcomes.

Studies and Year of Publication	Psychological and Somatic Outcomes		General Satisfaction			Overall Usability			
	Psychological Outcomes	Somatic Outcomes	Experience	Likeability	Perceived Benefit/ Helpfulness	Usability	Feasibility	Acceptability and Tolerance	Negative Effects
Brungardt et al., 2020 [34]									
Ferguson et al., 2020 [35]									
Johnson et al., 2020 [30]		*							
Lloyd & Haraldsdottir, 2021 [32]									
Niki et al., 2019 [23]	^	^	^§^						
Nwosu et al., 2021 [22]									
Perna et al., 2021 [31]	^†^	^†^							
Weingarten et al., 2019 [33]									

Note. The colours indicate positive findings (green), null findings (red) and variable findings (orange). Blank cells indicate the column header was not reported on. * Statistically significant improvement in lack of appetite based on 95% confidence interval (*p =* 0.279). ^ Significant improvements for pain (*p =* 0.018, *d* = 0.832), tiredness (*p =* 0.006, *d* = 0.804), drowsiness (*p =* 0.014, *d* = 0.590), lack of appetite (*p* = 0.043, *d* = 0.505) shortness of breath (*p =* 0.028, *d* = 0.681), depression (*p =* 0.008, *d* = 1.237), anxiety (*p =* 0.008, *d* = 0.788), well-being (*p =* 0.002, *d* = 1.175), fun (*p =* 0.003, *d* = 0.915) and happiness (*p =* 0.003, *d* = 0.962) in patients who had visited a memorable place. No significant improvement for participants who visited a place they had wanted to go but never visited. ^§^ Significant improvements for participants in pre-VR travel expectation and post-VR travel satisfaction for participants who had visited a memorable place (*p* = 0.041, *d* = 0.621). No significant improvement for participants who visited a place they had wanted to go but never visited. **^†^** No statistically significant change. However, personalised group appeared to experience largest reduction in tiredness, anxiety, and psychological well-being. Non-personalised group appeared to experience largest reduction in tiredness and drowsiness.

**Table 3:** Risk of bias assessment.

Studies and Year of Publication	Risk of Bias Score	Risk of Bias
Brungardt et al., 2020 [34]	4/9	High
Ferguson et al., 2020 [35]	6/9	Moderate
Johnson et al., 2020 [30]	6/9	Moderate
Lloyd & Haraldsdottir, 2021 [32]	4/9	High
Niki et al., 2019 [23]	6/9	Moderate
Nwosu et al., 2021 [22]	4/9	High
Perna et al., 2021 [31]	7/9	Low
Weingarten et al., 2019 [33]	2/9	High

### 3.5. Likeability

Likeability was mainly positive in the study by Brungardt et al. [34]; however, there were two reports of finding the experience “too boring”. Ferguson et al. [35] found that 56% (14/25) participants enjoyed the VR experience. Johnson et al. [30] reported that patients moderately liked the VR headset with a mean rating of 5.75 on a 10-point scale (with 1 representing least likability), while Lloyd and Haraldsdottir [32] found that overall, patient responses to VR were generally positive with some neutral responses and infrequent negative responses. Finally, Nwosu et al. [22] reported that all participants would like to use VR again, indicating likeability.

### 3.6. Perceived Benefit/Helpfulness

In the study by Johnson et al. [30], participants found the intervention moderately beneficial, with a mean rating of 4.42 on a 10-point scale, with 1 representing least beneficial. Lloyd and Haraldsdottir [32] found that some participants were able to find some fulfillment through the VR intervention in the form of having new experiences like visiting places they had never been. Others found benefit in the capacity to connect with the past and forget about their current situation during the VR experience. Similarly, the benefit of VR as a distraction from isolation was reported by Weingarten et al. [33]. Participants who started forgetting about their current situation also reported feeling calm and relaxed during the sessions, and that this feeling was maintained after the session had ended [32]. Staff evaluations conducted by Nwosu et al. [22] found that all respondents rated VR as helpful, with improvements in psychological well-being to be a benefit. 

### 3.7. Usability of VR

Several themes were identified relating to the overall usability of VR interventions in palliative care: general usability, feasibility, acceptability and tolerance, and negative effects.

#### 3.7.1. General Usability

Brungardt et al. [34] discovered an overall usability grade of 90% and reported that 53% of participants chose the highest satisfaction rating of 5/5 in reference to usability. Johnson et al. [30] reported that 67% (8/12) of participants found the VR easy to use and 58% (7/12) reported no difficulties in operation, and Nwosu et al. [22] reported some technical issues relating to setting up and charging the VR device.

#### 3.7.2. Feasibility

Brungardt et al. [34] found that overall, the VR intervention, including the creation of personalised soundtracks paired with a 360-degree VR environment, was feasible for patients in an intensive care unit and while patients were receiving treatments. The feasibility study by Ferguson et al. [35] found that VR shows promise for both patients and caregivers, while the study by Nwosu et al. [22] suggested feasibility and that VR was well received by patients, caregivers, and staff. Time and resourcing were identified as potential issues limiting feasibility. Perna et al. [31] reported that due to limited staffing resources, it took 20 months to identify and recruit 26 patients. Weingarten et al. [33], who designed a custom VR experience as a case study, found that time and scalability were two major considerations. Their research took two weeks to create a custom experience, and recommended creating a library of online and interactive resources with the ability for requests to be made for specific content to increase feasibility.

#### 3.7.3. Acceptability and Tolerance

Brungardt et al. [34] identified three themes regarding acceptability and tolerance. The first two were positive and included anticipation and appreciation. They reported that patients positively anticipated their participation in the intervention and appreciated the customised design. The third theme was acceptability and included varied responses, with some participants reporting not being able to see the images clearly. Although 8% (2/25) of participants had the headset removed due to an increase in pain score in Ferguson et al. [35], the VR experience was generally well tolerated. Johnson et al. [30] also concluded that their VR intervention was well tolerated by patients in palliative care, and Lloyd and Haraldsdottir [32] concluded that their intervention was acceptable for hospice patients in a hospice environment. Perna et al. [31] reported that no participants asked for the VR sessions to be stopped during the session, and 80% (20/25) of randomised participants met the criteria for full acceptability, defined as attending 4 out of 4 sessions. In addition, 4% (1/25) reported no acceptability, and 16% (4/25) reported partial acceptability. Finally, the case study by Weingarten et al. [33] tolerated the VR experience well with a limitation reported of a poor Wi-Fi connection. 

#### 3.7.4. Negative Effects

Brungardt et al. [34] reported that no participants described negative physical responses; however, there was one report of claustrophobia. Ferguson et al. [35] found that two participants experienced increased pain during the VR intervention and reported that the headset was heavy. They also reported that 20% (2/10) of caregivers reported worsened baseline symptoms at phone follow-up, with one participant experiencing increased crying and the other increased hallucinations. However, out of the 25 participants, 92% (23/25) reported no adverse events. Johnson et al. [30] reported that 17% (2/12) of participants complained of sore shoulders, which was attributed to repeated alignments of the HMD, with 83% (10/12) of participants reporting no adverse effects. No serious side effects were reported by Niki et al. [23]. Finally, Nwosu et al. [22] reported that 13% (2/15) of participants complained of minor problems, including heaviness of the headset, difficulty adjusting the head straps, and problems focusing on the image. In addition, staff reported problems relating to discomfort because of the headset and disorientation reported by some participants.

## 4. Discussion

The present review aimed to analyse and synthesise the available research on the use of VR in enhancing psychological and somatic outcomes, general satisfaction, and overall usability in palliative care patients. A narrative synthesis of the identified studies is presented to address these aims.

### 4.1. Enhancing Psychological Outcomes and Somatic Outcomes

The results on enhancing psychological and somatic outcomes varied across studies and research designs. As seen in Table 2, the four studies using qualitative methods reported positive findings [22,32,33,34], whereas the three studies with quantitative outcomes had mixed results [23,30,31]. These findings are promising, but there is currently still a lack of a strong evidence base for the effectiveness of VR for enhancing psychological and somatic outcomes in palliative care.

Most studies were feasibility or pilot studies with small sample sizes, and no firm conclusions can be drawn on the effectiveness of VR. However, there appears to be a pattern across the statistical results in which significant somatic and psychological symptom improvement were observed where the VR experience was made more personally meaningful by providing a tailored experience. Several mechanisms may underlie these findings.

The first possible mechanism that can help explain the improvements in psychological symptomatology in studies that provided a personally meaningful experience is the reminiscence concept. It has previously been demonstrated that reminiscence can provide psychological benefits [36]. This intervention is primarily used with older adults who find sharing autobiographical memories a meaningful activity; its goal is to activate the social functions of reminiscence to enhance positive feelings and a sense of identity [36]. This mechanism is supported by Niki et al. [23], who noted that memories of the destination visited may have influenced the positive results. Therefore, VR could be a useful tool to facilitate reminiscence sessions in a palliative care setting because of its ease of use, portability, and ability to immerse the user [16,17]. Future research should consider using VR to facilitate other evidence-based psychological therapies such as dignity therapy to enhance outcomes in palliative care patients.

While using VR to facilitate other evidence-based psychological therapies in palliative care is exciting, the concepts of distraction and relaxation may also contribute to improvements. There were studies reviewed here that contained feedback from participants relating to a sense of being removed from their current situation during the VR intervention, which may have relieved somatic and psychological symptoms [32,33,34]. The effectiveness of VR distraction for pain is well established [37]. However, results may be influenced by patient population and type of pain [38,39]. This review included participants with different conditions and at varying stages of disease progression, which may have contributed to the mixed results. Although the results are inconclusive at this stage, using VR as a tool for evidence-based therapies in palliative care is promising. 

### 4.2. General Satisfaction with VR

The only study that assessed satisfaction quantitatively found significant improvements in the VR experience only in participants who visited a memorable place [23], similar to the somatic and psychological outcomes also reported by Niki et al. [23]. Qualitative feedback in the remaining studies found that patients appeared to be generally satisfied with the VR intervention. 

In line with the psychological and somatic outcomes, the patients’ general satisfaction with their VR experience seems to differ depending on the offered intervention. For example, participants that were given a choice of their VR experience appeared to describe the experience more positively [22,32] than those who were offered a 3.5-min looped beach scene [35]. Furthermore, the content quality and the HMD device may influence the user’s immersion and experience in the virtual environment. For example, high-quality content combined with HMD hardware that can display content at a high resolution may provide the user with a better experience [40]. This is relevant here because various content and VR technologies were used across the studies considered for this review (see Table 1), and may have influenced the VR experience.

Another variable that may influence the VR experience is the interactivity of the intervention offered. Interactivity and enjoyment are positively correlated [41]. The ability to interact in the virtual environment was requested by participants in Brungardt et al. [34]. Furthermore, Johnson et al. [30] stated that providing interaction may increase therapeutic benefits. However, there is a potential problem with interactivity in a palliative care population for those with limited mobility, as VR controllers must be used to interact with the virtual environment. Some applications now offer voice control which could help with this problem in the future. While consistency between research will always be challenging to achieve, the quality and interactivity of VR to improve the experience and general satisfaction should be considered in future research.

### 4.3. Usability of VR

Overall, studies found the VR intervention usable, feasible, and acceptable. Some isolated negative feedback could partially be explained by fitment of the HMD or dissatisfaction with the intervention [34,35]. In addition, technical issues and time to create content were reported [22,33]. The increasing availability of content and current generation HMDs that are lighter with improved head straps should mitigate some of these issues reported.

The overall acceptability of the VR interventions is consistent with previous studies [5,42]. However, the use of VR in a palliative care population requires extra care due to the presentation of the patients, many of whom are often older or frail. The study by Johnson et al. [30] provided a 5 min training session on the use of the HMD and hand controllers and stated that multiple VR sessions might allow participants to become more proficient. This population consists of patients with diverse needs and abilities, and some will likely need more instruction or assistance than others. This may have implications for feasibility if trained individuals are required to teach the user how to set up and properly operate the VR device. Current and continual improvement of HMD technology will assist with improving usability that may also make VR more appealing for patients in a palliative care setting.

Although the novelty of the VR experience can be seen as a benefit to recruitment, the question of what happens when the novelty of the experience wears off and participants are no longer interested in engaging remains. Indeed, Johnson et al. [30] had one participant report that they were happy that they now knew what it was like to use a VR headset. However, in the study by Ferguson et al. [35], one participant stated, “it’s a one-time experience; you don’t need it twice” (p. 813).

There were few adverse or minor negative effects reported from HMD use. An increase in crying and hallucinations in two participants at 3–5 h following the intervention reported by Johnson et al. [30] is of most concern. This effect highlights the importance of monitoring for negative side effects after a VR experience in the hours immediately following the intervention. Indeed, a 2015 study found that visual hallucinations occurred in six of 23 patients with Parkinson’s disease who had participated in an immersive VR protocol [43]. Therefore, extra care and follow-up are needed when using VR interventions in patients with neurodegenerative diseases such as Parkinson’s and dementia. Furthermore, study protocols should include measures of side effects to monitor for symptoms during and after VR sessions, as there were studies in this review that did not report how well tolerated VR was.

### 4.4. Limitations

The studies in this review considered many different palliative care patients with a range of diagnoses and stages of disease progression. However, limitations of the studies considered in this review include heterogeneity between the measures and the small sample sizes in each study. With only three studies using a validated symptom measure, the majority of the data in this review were qualitative, and limited statistical evidence was available. No RCTs were found, and no studies utilised true control groups. Furthermore, a wide variety of VR technologies were used, including older systems using smartphones inserted into the HMD. Finally, seven of the eight included studies were judged as having a moderate or high risk of bias.

## 5. Conclusions and Future Directions

The use of VR has promise for addressing a range of symptoms in palliative care patients. Most studies included in this review showed a positive effect on symptoms of interest with few adverse effects. The promising results found in the included studies provide the groundwork for further investigating the use of VR in palliative care. Constantly increasing the availability of VR content and improvements in HMD hardware will assist in improving the VR experience and enhancing outcomes.

Recruitment of patients in palliative care is challenging. Barriers include a lack of interest from participants, the nature of their illness, and the identification of participants that meet inclusion criteria [44]. Given the need for adequately powered RCTs in this area in the future, the feasibility of recruitment for larger studies needs to be considered. Investigating ways to efficiently and ethically utilise VR and identifying if it can continue to be meaningful and enjoyable over time will be beneficial for recruiting in future research. This may be achieved by the improvements in HMD technology combined with providing meaningful activities.

Future research should consist of adequately powered RCTs to consider multiple variables to enhance outcomes in this population. The opportunity for VR interventions meaningful to participants will be of utmost importance, as it was apparent in this review that meaningful activities were associated with better outcomes. Furthermore, VR dosage in this population is largely unknown and needs further investigation, and may vary depending on the type of intervention. For example, reminiscence-based approaches may require more sessions compared with interventions based on distraction. Regardless, it is clear that VR has a major role to play across a range of health care settings, including in palliative care patients. 

## Data Availability

The study protocol can be found on https://osf.io/5jqux (accessed on 23 June 2022). The data are available from the corresponding author upon reasonable request.
